# Problem-solving and social support appraisals in the cross-sectional association between social media integration and PCL-5 symptom severity

**DOI:** 10.1371/journal.pone.0362802

**Published:** 2026-11-12

**Authors:** Tyrone B. Pretorius, Anita Padmanabhanunni

**Affiliations:** 1 Department of Psychology, University of the Western Cape, Cape Town, South Africa; 2 Monash University, Melbourne, Australia; Hochschule Niederrhein - Campus Mönchengladbach: Hochschule Niederrhein - Campus Monchengladbach, GERMANY

## Abstract

The present study investigated the relationship between social media integration and PTSD-consistent symptomatology among South African university students, with a specific focus on the roles of problem-solving appraisal and perceived social support. Participants were 491 students who completed the Social Media Use Integration Scale, the Problem-Solving Inventory-20, the Multidimensional Scale of Perceived Social Support, and the Post-Traumatic Stress Disorder Checklist for DSM-5 (PCL-5). Data were analysed using descriptive statistics, correlations, and mediation and moderation analyses with PROCESS. Social media integration was positively associated with overall PTSD-consistent symptomatology and all symptom clusters measured by the PCL-5. Problem-solving appraisal was negatively associated with PCL-5 symptom severity. It partially mediated the relationship between social media integration and PCL-5 symptom severity. Perceived social support showed a more selective pattern of association, with higher support linked to lower negative alterations in cognition and mood, as well as hyperarousal. In addition, social support moderated the relationship between social media use and PCL-5 symptom severity, avoidance, negative alterations in cognition and mood, and hyperarousal. These findings suggest that greater social media integration is associated with higher PCL-5 symptom severity, whereas problem-solving appraisal and perceived social support serve as important protective factors. Interventions aimed at strengthening coping appraisals and supportive social networks may help reduce trauma-related distress among young adults in the context of frequent social media use.

## Introduction

Social media use has become a pervasive aspect of the daily lives of adolescents and young adults, and now constitutes an important medium through which socialisation, interpersonal relatedness, and identity formation occur [1]. As such, it has become closely integrated with normative developmental processes during adolescence and emerging adulthood. The increasing centrality of social media in the lives of young people has generated growing research interest in its psychological and behavioural consequences [2–4].

Existing evidence indicates that social media use may contribute to a range of adverse mental health outcomes. In particular, the literature has predominantly focused on depression, anxiety, stress, loneliness, and physical health-related outcomes such as sleep problems and insomnia [4,5]. By contrast, less attention has been directed towards the relationship between social media use and post-traumatic stress disorder (PTSD). The current study was undertaken in the South African context, where research has documented a high prevalence of trauma exposure and PTSD among the student population [6–8]

Existing research examining PTSD in relation to social media use has generally developed along three overlapping lines. First, a growing body of work has focused on vicarious or indirect trauma exposure through social media [9,10]. In this literature, repeated engagement with posts, images, videos, and online discussions about disasters, violence, or socio-political unrest is viewed as a pathway through which trauma-related distress may emerge even in the absence of direct exposure. Second, another line of inquiry has examined the possibility that social media may exacerbate existing PTSD symptoms by exposing trauma survivors to distressing reminders, emotionally charged content, or information overload [11,12]. Third, social media has also been conceptualised as an avoidance-based or distraction-oriented coping mechanism. Some individuals with PTSD appear to use social media to distract from thoughts about the traumatic event and to manage distress in the short term; however, this strategy may be maladaptive over time [13]. This raises questions about how individuals perceive their capacity to manage distress and provides a rationale for considering appraisals of personal coping capabilities.

Cognitive appraisals are widely recognised as central to psychological adjustment and have been consistently implicated in the development and maintenance of a range of mental health difficulties [6,14,15]. In relation to PTSD, most cognitive theories emphasise the role of negative appraisals in the onset and persistence of symptoms, particularly maladaptive beliefs about the self, the world, and the consequences of traumatic experiences. These negative appraisals heighten perceptions of threat, maintain distress, and contribute to the persistence of symptoms over time [16,17]. In this context, the present study sought to extend the literature by examining whether appraisals of problem-solving ability and social support are relevant cognitive mechanisms linking social media integration and symptom severity measured using the Posttraumatic Stress Disorder Checklist for DSM-5 (PCL-5), with particular attention to the roles of problem-solving appraisal and perceived social support.

A previous analysis of the same dataset examined associations between social media integration and several indicators of psychological distress, including PCL-5 scores, with smartphone addiction as a proposed intervening variable [18]. The present study extends that work by shifting the focus from problematic smartphone engagement to appraisals of personal coping resources and perceived interpersonal support. Specifically, this study examines cross-sectional indirect associations between social media integration and PCL-5 total and symptom-domain scores through problem-solving appraisal and perceived social support. Additional exploratory analyses examine whether these associations vary according to perceived social support. Although the studies share participants and some measures, the present analysis addresses a different question concerning how cognitive appraisals and perceived support relate to the association between social media integration and PCL-5 symptom severity.

Problem-solving appraisal refers to an individual’s subjective evaluation of their capacity to manage and resolve problems effectively [19]. It reflects how people perceive their own problem-solving abilities, rather than how well they actually perform when confronted with a problem. In this sense, problem-solving appraisal concerns confidence, perceived control, and beliefs about one’s effectiveness in approaching everyday challenges [19]. It differs from actual problem-solving, which refers to the observable cognitive and behavioural processes involved in identifying, analysing, and addressing a problem.

Perceived social support complements problem-solving appraisal by directing attention to interpersonal resources. Whereas problem-solving appraisal concerns individuals’ confidence in their ability to manage difficulties, perceived social support concerns their belief that others are available to provide assistance, understanding, and reassurance [20]. The distinction is important: individuals may feel confident in their own problem-solving abilities yet perceive little support from others, or perceive strong support yet doubt their ability to manage challenges independently. Examining both constructs, therefore, allows consideration of related yet distinct appraisals of the resources available for managing distress.

These distinctions are relevant when considering social media integration. Social media may provide opportunities to maintain relationships and seek assistance, but emotional attachment to a platform does not establish the quality or adequacy of the support experienced through it [4,21,22].

This study is grounded in Lazarus and Folkman’s theory of stress and coping [23]. According to this framework, individuals first engage in a primary appraisal of whether an encounter is benign or threatening, followed by a secondary appraisal of whether they possess the personal and social resources needed to manage it. Problem-solving appraisal can be understood as an aspect of secondary appraisal because it reflects individuals’ perceptions of their ability to manage difficulties. Perceived social support similarly concerns secondary appraisal, reflecting beliefs about the availability of assistance, understanding and reassurance from others. Together, these constructs capture complementary perceptions of personal and interpersonal coping resources that may be relevant to responses to stressors, including potentially traumatic experiences. This provides a theoretical rationale for examining both constructs in relation to social media integration and PCL-5 symptom severity.

## Materials and methods

### Participants and procedure

Participants (*n* = 491) were students at a university in the Western Cape province of South Africa. An electronic questionnaire was constructed using Google Forms, and the university Registrar randomly selected 4000 students and emailed them a link and an invitation to participate. Participation was voluntary, and data collection occurred from 4 March 2024–30 October 2024.

A total of 491 students consented to participate and submitted completed questionnaires, corresponding to a response rate of 12.3%. Information regarding the number of students who opened the invitation was not recorded. Participants could access the survey only after providing informed consent on the survey landing page.

A sensitivity analysis, conducted with the package “pwr2ppl” [24] in R software [25], found that with two mediators and medium effect sizes, a sample of 180 would provide 80.2% power for mediator one and 80.6% power for mediator two. The actual sample of 491 achieved 99.3% and 99.8% power for mediators one and two, respectively. For the moderation analysis, the sensitivity analysis indicated that, for a medium R2 change (from the full regression model to a model that excludes the interaction term), a sample size of 226 would provide 80.2% power, whereas the actual sample size of 491 would achieve 98.6% power.

The majority of the sample were women (64.8%), and the mean age was 21.22 years (*SD* = 3.52; range = 17–44). Only 7.5% of the sample were postgraduate students, and the rest were undergraduate (92.5%). The participants came from all nine provinces in South Africa, but the majority were drawn from the Eastern (27.5%) and the Western Cape (32.8%). The participants resided in both urban (64.8%) and rural areas (35.2%)

### Measures

The participants completed the following instruments: the Social Media Use Integration Scale (SMUIS) [26], the Problem-Solving Inventory-20 (PSI-20) [27], the Multidimensional Scale of Perceived Social Support (MSPSS) [28], and the Post-Traumatic Stress Disorder Checklist for DSM-5 (PCL-5) [29].

The SMUIS is a 10-item scale that assesses the extent to which social media is incorporated into individuals’ everyday routines and social interactions, as well as their emotional connection to social media. It therefore captures the psychological and social integration of social media into daily life rather than objective behavioural indicators such as the frequency of platform access, time spent on social media, or duration of use. The original SMUIS was developed and validated using Facebook users, and its 10 items were consequently worded with reference to Facebook [26]. However, the developers intended the scale to be adaptable to other social media services by replacing “Facebook” with the relevant social media referent. For the present study, references to “Facebook” and “Facebook account” were replaced with “social media” and “social media account(s)”, respectively, to assess participants’ experiences across social media platforms. An example of an item of the SMUIS is “I get upset when I can’t log on to social media (e.g., Instagram, Facebook, TikTok, etc.).” No other changes were made to the substantive content of the items or the six-point response scale.

The SMUIS is scored on a six-point scale ranging from 1 (strongly disagree) to 6 (strongly agree), and higher scores reflect greater integration of social media use. The authors of the scale reported a reliability coefficient of.91 for SMUIS scores and provided evidence for convergent and discriminant validity [26]. In South Africa, Maree [30] reported reliability coefficients of.89 for SMUIS scores when used in relation to Facebook and.79 when it was used in relation to LinkedIn.

The PSI-20 is a 20-item shortened version of the original 35-item PSI [19] and is used to assess participants’ self-appraisal of their effectiveness as problem-solvers. It is scored on a six-point scale that ranges from 1 (strongly disagree) to 6 (strongly agree). An example item of the PSI-20 is “I make decisions, and I am happy with them later.” In the original PSI-35, items were scored such that high scores reflected perceptions of ineffective problem-solving. In the current study, the items were scored such that high scores reflected perceptions of effective problem-solving. Pretorius and colleagues [27] reported satisfactory estimates of internal consistency for PSI-20 scores (α and ω = .88). They found that the shortened version of the PSI had relationships with indices of psychological distress similar to those of the original PSI.

The MSPSS is a 12-item measure of the adequacy of perceived social support received from family, friends, and a significant other. It is scored on a seven-point scale ranging from 1 (very strongly disagree) to 7 (very strongly agree), with higher scores indicating greater perceived social support. An example item on the MSPSS is “I can talk about my problems with my family.” The authors of the MSPSS reported an internal consistency estimate of.88 and provided evidence of construct validity. A study with South African youth reported a reliability coefficient of.86 for MSPSS scores [31].

The PCL-5 consists of 20 items that measure the 20 DSM-5 symptoms of PTSD. The participants respond to the 20 items using a five-point scale that ranges from 0 (not at all) to 4 (extremely), and high scores on the PCL-5 reflect greater severity of PTSD symptoms. The PCL-5 provides a total score and scores for the four DSM-5 symptom clusters: re-experiencing, avoidance, negative alterations in cognition and mood, and hyperarousal. Re-experiencing involves involuntary and distressing memories of the traumatic event (example item: “How much were you bothered by repeated, disturbing dreams of the stressful experience?”). Avoidance refers to efforts to avoid distressing thoughts, memories, feelings, or external reminders associated with the trauma (example item: “How much were you bothered by avoiding memories, thoughts, or feelings related to the stressful experience?”). Negative alterations in cognition and mood refer to persistent and exaggerated negative beliefs about the self or the world, and to the distorted blaming of self and others (example item: “How much were you bothered by blaming yourself or someone else for the stressful experience or what happened after it?”). Hyperarousal refers to Irritability or anger outbursts, reckless or self-destructive behaviour, hypervigilance, difficulty concentrating, and sleep disturbances (example item: “How much were you bothered by irritable behaviour, angry outbursts, or acting aggressively?”). The scale developers reported estimates of internal consistency for the PCL-5 scores of.94 and.95 in two different studies and provided evidence of convergent and discriminant validity in both studies [29]. In South Africa, Padmanabhanunni and Pretorius [32] reported similar strong estimates of internal consistency for the PCL-5 (α and ω = .94).

### Ethics

The Biomedical Science Research Ethics Committee of the University of the Western Cape provided ethical approval (Ethics reference: BM23/10/8, 4 December 2023), and the study was conducted in accordance with the Declaration of Helsinki. This was a two-phase study. The first phase involved collecting baseline data on mental health outcomes. For the second phase, which entailed collecting comparative data at other institutions and evaluating the efficacy of a digital mental health intervention, the ethics committee extended ethical approval until July 2026. Participation was voluntary, and participants provided informed consent on the landing page of the electronic link. No incentives were offered for participation.

The electronic questionnaire was administered through Google Forms using the university’s institutional Google Workspace account. The use of Google Forms for data collection was explicitly specified in the research proposal, which was reviewed and approved by the institutional ethics committee. Access to the form and its response data was restricted to the researchers through password-protected institutional accounts and the university’s authentication and access-control procedures. The survey link allowed participants to submit responses but did not provide access to others’ responses. No identifying information was collected. Following data collection, the response data were downloaded and stored on a password-protected, access-controlled university system. Access was limited to authorised members of the research team. The data were managed and retained in accordance with the approved ethics protocol and the university’s data-management requirements.

The present study forms part of a broader project in which participants completed several measures of digital engagement, psychological resources, and mental health. The same cross-sectional dataset has therefore supported several publications addressing distinct research questions. Previous analyses of this dataset examined: cross-sectional indirect associations between smartphone addiction and indices of psychological distress through problem-solving appraisal [33]; the interaction between perceived stress and perceived social support in relation to PCL-5 scores [34]; and associations between smartphone addiction and PCL-5 scores, with resilience examined as a moderator [35]. These studies did not examine the specific model tested in the present manuscript.

One previous publication used the SMUIS and examined associations between social media integration and several indices of psychological distress, with smartphone addiction as the proposed intervening variable [18]. The present manuscript addresses a different research question by examining cross-sectional indirect associations between social media integration and PCL-5 total and symptom-domain scores through problem-solving appraisal and perceived social support, as well as exploratory conditional associations involving perceived social support. It does not reanalyse smartphone addiction or resilience as explanatory variables.

### Data analysis

All questionnaire items were configured as forced-choice, and participants could not submit the questionnaire with any missing responses. Consequently, there were no missing data for the core variables. However, demographic questions were not marked as mandatory, and one participant did not respond to the age question; age was used as a covariate. For the other covariate, 10 participants indicated their gender as “other” and were excluded from the analyses. Thus, the final sample size was n = 480 for each mediation and moderation model. IBM SPSS for Windows version 31 (IBM Corp., Armonk, NY, USA) was used for all analyses. This included the intercorrelations between study variables (Pearson’s *r*), descriptive statistics (means and standard deviations), distribution indices (skewness and kurtosis), and estimates of internal consistency of scale scores (Cronbach’s alpha). The correlation coefficients were evaluated for statistical significance and Cohen’s effect sizes, where.10 is a small effect,.30 a medium effect, and.50 a large effect [36]. Skewness and kurtosis were used to assess the distribution of data, and if these indices were between −2 and +2, the data were approximately normally distributed [37]. In terms of the reliability of the scores of instruments, an alpha greater than 0.70 is typically regarded as acceptable for research purposes [38].

The role of a third variable in the relationship between two other variables can be conceptualised as moderating or mediating that relationship [39]. Since a variable can serve as both a moderator and a mediator [40], we conducted exploratory mediation and moderation analyses for social support and problem-solving appraisal.

Mediation analysis was conducted using the PROCESS macro in SPSS (model 4). In the mediation model, social media integration was the independent variable, PCL-5 symptom severity was the dependent variable, and problem-solving appraisal and social support were used as parallel mediators. The statistical significance of the indirect effects of social media use via problem-solving appraisal and social support was evaluated using bootstrapped 95% confidence intervals (5000 samples); if these confidence intervals did not include zero, the effect was considered statistically significant.

Exploratory moderation analyses (model 1) were conducted to examine whether the cross-sectional associations between social media integration and PCL-5 total and symptom-domain scores varied according to perceived social support and problem-solving appraisals. Social media integration, perceived social support, and problem-solving appraisal were mean-centered in the respective analyses to facilitate interpretation of the regression coefficients, and their product was entered as an interaction term alongside both constituent predictors. Statistically significant interactions were examined by estimating conditional associations and plotting regression lines at the mean and at one standard deviation below and above the mean of perceived social support or problem-solving appraisal. These analyses were interpreted as exploratory tests of conditional associations. To account for multiple testing, an online false discovery rate calculator based on the Benjamini-Hochberg method [41] was used to provide adjusted p-values.

T-tests were used to examine gender differences in the dependent variables, and correlational analyses were used to examine the relationships between age and the dependent variables. When significant gender differences or age-related relationships were observed, those variables were included as covariates in the mediation and moderation analyses.

## Results

Reported in Table 1 are the intercorrelations between study variables, descriptive statistics, distribution indices, and estimates of internal consistency for the scores of measures used in the study.

**Table 1:** Intercorrelations among study variables, descriptive statistics, distribution indices, and reliabilities of measure scores.

	1	2	3	4	5	6	7	8
1. Social media integration	−−							
2. Problem-solving appraisal	−.16^**^	−−						
3. Social support	.09^*^	.13^*^	−−					
4. PCL-5 symptom severity	.35^**^	−.28^**^	−.08	−−				
5. Re-experiencing	.29^**^	−.23^**^	−.02	.87^**^	−−			
6. Avoidance	.28^**^	−.16^**^	.00	.80^**^	.71^**^	−−		
7. Negative alterations have	.33^**^	−.29^**^	−.13^*^	.93^**^	.69^**^	.68^**^	−−	
8. Hyperarousal	.33^**^	−.24^**^	−.09^*^	.90^**^	.67^**^	.61^**^	.79^**^	−−
Mean	32.88	82.72	58.01	32.57	7.99	3.63	11.52	9.43
*SD*	10.69	11.00	17.11	18.62	5.50	2.49	7.18	5.81
Range	10-60	41-116	12-84	0-80	0-20	0-8	0-28	0-24
Skewness	0.40	−0.47	−0.65	0.12	0.28	0.14	0.18	0.21
Kurtosis	−0.51	1.07	−0.32	−0.61	−0.86	−1.03	−0.70	−0.63
Alpha	.87	.74	.93	.94	.89	.81	.88	.83

* p < .05, ** p < .001.

The skewness values in Table 1 ranged from −0.65 to 0.40, while the kurtosis values ranged from −1.03 to 1.07; thus, both indices provide evidence that the data were approximately normally distributed. The estimates of internal consistency in Table 1 all exceeded.70 and ranged between.74 and.94.

Table 1 further indicates that social media integration was significantly and negatively associated with problem-solving appraisal (*r* = −.16, *p* < .001, small effect size), and significantly and positively associated with social support (*r* = .09, *p* = .04, small effect size). Therefore, perceptions of effective problem-solving were associated with lower social media integration, whereas higher social support was associated with higher social media integration. Social media integration was also significantly positively associated with PCL-5 symptom severity (*r* = .35, *p* < .001, medium effect size) and all the symptom clusters, namely PCL-5 re-experiencing (*r* = .29, *p* < .001, small effect size), PCL-5 avoidance (*r* = .28, *p* < .001, small effect size), PCL-5 negative alterations in cognition and mood (*r* = .33, *p* < .001, medium effect size), and PCL-5 hyperarousal (*r* = .33, *p* < .001, medium effect size). The cross-sectional associations indicate that higher levels of social media use were associated with greater PCL-5 symptom severity, re-experiencing, avoidance, negative alterations in cognition and mood, and hyperarousal.

Problem-solving appraisal was negatively related to PCL-5 symptom severity (*r* = −.28, *p* < .001, small effect size), re-experiencing (*r* = −.23, *p* < .001, small effect size), avoidance (*r* = −.16, *p* < .001, small effect size), negative alterations in cognition and mood (*r* = −.29, *p* < .001, small effect size), and hyperarousal (*r* = −.24, *p* < .001, small effect size). These results demonstrate that appraisals of effective problem-solving were associated with lower levels of PCL-5 symptom severity, re-experiencing, avoidance, negative alterations in cognition and mood, and hyperarousal.

At the zero-order level, perceived social support was not significantly associated with the PCL-5 total score, re-experiencing, or avoidance. Small negative associations were observed with negative alterations in cognition and mood (*r* = −.13, *p* = .01, small effect size) and hyperarousal (*r* = −.09, *p* = .04, small effect size). Thus, the zero-order correlations did not indicate a general association between perceived social support and PCL-5 symptom severity; rather, the significant associations were small and limited to two PCL-5 symptom domains.

The results of the t-tests indicated that women reported higher levels of PCL-5 symptom severity ($\overset{―}{X}$ = 34.59, *SD* = 18.54), re-experiencing ($\overset{―}{X}$ = 8.52, *SD* = 5.85), avoidance ($\overset{―}{X}$ = 3.94, *SD* = 2.54), negative alterations in cognition and mood ($\overset{―}{X}$ = 12.24, *SD* = 7.03), and hyperarousal ($\overset{―}{X}$ = 9.89, *SD* = 5.84) than men (PTSD: $\overset{―}{X}$ = 28.85, *SD* = 17.87, *t* = 3.41, *p* < .001; re-experiencing: $\overset{―}{X}$ = 6.93, *SD* = 5.00, *t* = 3.16,

*p* = .002; avoidance: $\overset{―}{X}$ = 3.04, *SD* = 2.28, *t* = 3.95, *p* < .001; negative alterations in cogni*t*ion and mood: $\overset{―}{X}$ = 10.06, *SD* = 7.07, *t* = 3.22, *p* = .001; hyperarousal: $\overset{―}{X}$ = 8.56, *SD* = 5.62, *t* = 2.39, *p* = .017). Correlational analyses showed that age was significantly negatively associated with PCL-5 symp*t*om severity (*r* = −.14, *p* = .001, small effect size), re-experiencing (*r* = −.11, *p* = .019, small effect size), avoidance (*r* = −.13, *p* = .004, small effect size), nega*t*ive alterations in cognition and mood (*r* = −.15, *p* = .001, small effect size), and hyperarousal (*r* = −.12, *p* = .007, small effect size). Accordingly, gender and age were included as covariates in the mediation and moderation analyses.

Table 2 presents the direct effects from the mediation analysis.

**Table 2:** The direct effects of social media use, problem-solving appraisal, and social support on PCL-5 symptom severity in the mediation analysis.

Effect	B	SE	95% CI		β
			LL	UL	
Social media integration → PCL-5 symptom severity	.56	.08	.41	.71	.32***
Social media integration → Re-experiencing	.12	.02	.08	.17	.24***
Social media integration → Avoidance	. 05	.01	.03	.07	.23***
Social media → Negative alterations	.19	.03	.13	.24	.28***
Social media → Hyperarousal	.16	.02	.11	.21	.30***
Problem-solving appraisal → PCL-5 symptom severity	−. 33	.07	−. 47	−. 19	−. 19***
Problem-solving appraisal → Re-experiencing	−. 08	.02	−. 13	−. 04	−. 17***
Problem-solving appraisal → Avoidance	−. 02	.01	−. 04	−. 00	−.09*
Problem-solving appraisal → Negative alterations	−. 14	.03	−. 19	−. 08	−. 21***
Problem-solving appraisal → Hyperarousal	−.09	.02	−. 13	−. 04	−. 16***
Social support → PCL-5 symptom severity	−.09	.05	−.18	−.00	−. 09*
Social support → Re-experiencing	−.01	.01	−. 04	.02	−.02
Social support → Avoidance	−.00	.01	−. 02	.01	−. 02
Social support → Negative alterations	−.05	.02	−.09	−.02	−. 12**
Social support → Hyperarousal	−.03	.02	−.06	−.00	−.09*

*p < .05, **p < .01, ***p < .001.

Table 2 reflects that the direct effects of social media integration on PCL-5 symptom severity (β = .32, *p* < .001), re-experiencing (β = .24, *p* < .001), avoidance (β = .23, *p* < .001), negative alterations in cognition and mood (β = .28, *p* < .001), and hyperarousal (β = .30, *p* < .001) were all positively significant in the presence of the mediator. Problem-solving appraisal also had significant negative direct effects on PCL-5 symptom severity (β = −.19, *p* < .01), re-experiencing (β = −.17, *p* < .001), avoidance (β = −.09, *p* = .01), negative alterations in cognition and mood (β = −.21, *p* < .001), and hyperarousal (β = −.16, *p* < .001). Conversely, social support only had significant negative direct effects on PCL-5 symptom severity (β = −.09, *p* = .03), negative alterations in cognition and mood (β = −.12, *p* = .00), and hyperarousal (β = −.09, *p* = .02). All pathways in Table 2 remained significant after applying the false discovery rate procedure except for the direct effects of problem-solving appraisal on avoidance (adjusted *p*-value = 0.053), and the direct effects of social support on PCL-5 symptom severity (adjusted *p*-value = 0.053).

Although perceived social support was not significantly correlated with the PCL-5 total score at the zero-order level, it showed a small negative adjusted association after social media integration and problem-solving appraisal were included in the parallel mediation model (β = −.09, *p* = .046). Negative adjusted associations were also observed for negative alterations in cognition and mood (β = −.12, *p* = .004), and hyperarousal (β = −.09, *p* = .032). These coefficients represent the cross-sectional associations between perceived social support and the outcomes, after accounting for the other variables in the model; they are therefore not equivalent to the unadjusted zero-order correlations reported in Table 1. However, the adjusted Benjamini-Hochberg p-value indicated that the association between social support and PCL-5 symptom severity was an artifact of multiple testing.

Table 3 depicts the indirect effects obtained in the mediation analysis.

**Table 3:** The indirect effects of social media integration on PCL-5 symptom severity through problem-solving appraisal and social support in the mediation analysis.

Pathway	Indirect effects					Total effects^1^		
	B	SE	95% CI		β	B	95% CI	
			LL	UL			LL	UL
Social media integration → Problem-solving appraisal → PCL-5 symptom severity	.044	.020	.007	.089	.025**	.556**	.407	.706
Social media integration → Problem-solving appraisal → Re-experiencing	.011	.006	.002	.024	.022**	.133**	.087	.178
Social media integration → Problem-solving appraisal → Avoidance	.003	.002	.000	.007	.012**	.055**	.035	.076
Social media integration → Problem-solving appraisal → Negative alterations	.019	.008	.004	.037	.028**	.200**	.142	.258
Social media integration → Problem-solving appraisal → Hyperarousal	.012	.006	.002	.025	.021**	.169**	.121	.216
Social media integration → Social support → PCL-5 symptom severity	−.011	.009	−.032	.005	−.006			
Social media integration → Social support → Re-experiencing	−.001	.002	−.005	.003	−.002			
Social media integration → Social support → Avoidance	.000	.001	−.002	.002	−.002			
Social media integration → Social support → Negative alterations	−.006	.004	−.016	.002	−.009			
Social media integration → Social support → Hyperarousal	−.004	.003	−.011	.002	−.007			

^1^The total effects listed under the problem-solving appraisal pathway include the corresponding social support pathway.

**Statistically significant effects as 95% CI does not include zero.

Table 3 shows a significant indirect effect of social media integration on the PCL-5 total score through problem-solving appraisal (β = .025, 95% CI [.007,.089]). Significant indirect associations through problem-solving appraisal were also observed for social media integration on re-experiencing (β = .022, 95% CI [.002,.024]), avoidance (β = .012, 95% CI [.000,.007]), negative alterations in cognition and mood (β = .028, 95% CI [.004,.037]), and hyperarousal (β = .021, 95% CI [.002,.025]. These results provide evidence for the mediating role of problem-solving appraisal in the relationship between social media integration and PCL-5 symptom severity as well as across all PCL-5 symptom clusters. However, since the direct effects of social media integration and PCL-5 symptom severity (and the PCL-5 symptom clusters) were significant in the presence of problem-solving appraisal in the mediation model, as indicated in Table 2, it would indicate that problem-solving appraisal only partially mediated the effects of social media integration on PCL-5 symptom severity.

In contrast, none of the indirect associations through perceived social support was statistically significant because all corresponding bootstrap confidence intervals included zero. This finding should be distinguished from the small negative adjusted associations between perceived social support and selected PCL-5 outcomes reported in Table 2. It should also be distinguished from the zero-order results in Table 1, where perceived social support was not significantly associated with the PCL-5 total score but showed small negative associations with negative alterations in cognition and mood as well as hyperarousal. The results therefore do not support a cross-sectional indirect association through perceived social support. Fig 1 visually presents the mediation model.

**Fig 1:**
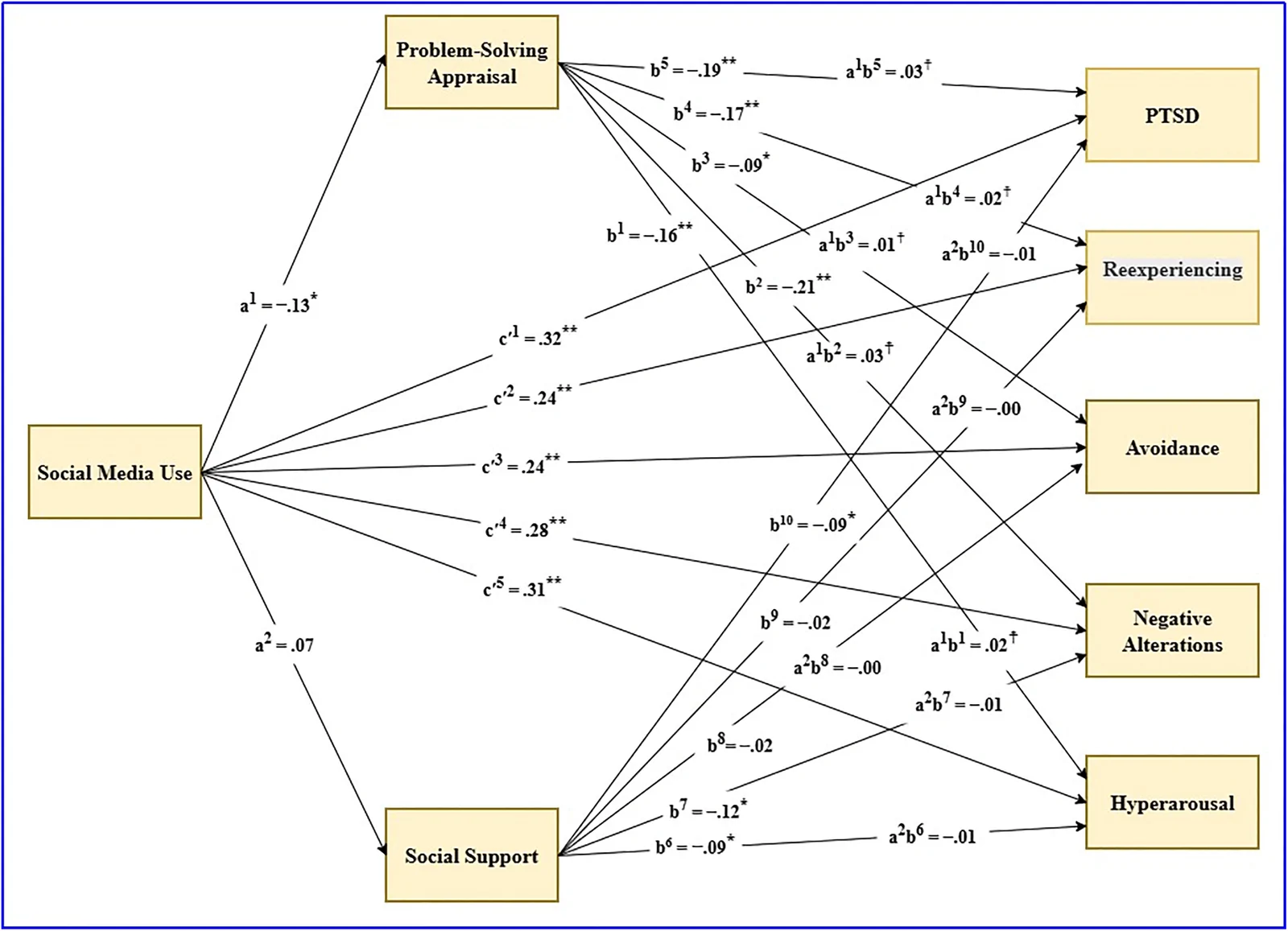
Visual presentation of the parallel mediating roles of problem-solving appraisal and social support in the relationship between social media integration and PCL-5 symptom severity. All regression coefficients are standardised. a^1^ and a^2^ = the cross-sectional association between the predictor and the two mediators, c′^1^ to c′^5^ = the direct effects of the predictor on the dependent variables in the presence of the mediators, b^1^ to b^10^ = the direct effects of the two mediators on the dependent variables, a^1^b^1^ to a^1^b^5^ = the indirect effects of the predictor on the dependent variables via mediator one, a^2^b^6^ to a^2^b^10^ = the indirect effects of the predictor on the dependent variables via mediator two. * p < .05, ** p < .001, ^☨^statistical significance established with 95% Confidence Intervals.

The results of the moderation analysis indicated that social support significantly moderated the relationships between social media integration, on the one hand, and PCL-5 symptom severity (B = −.009., *p* = .024), avoidance (B = −.001, *p* = .038), negative alterations in cognition and mood (B = −.004, *p* = .018), and hyperarousal (B = −.003, *p* = .029), on the other hand. There was no significant interaction between social media integration and social support regarding re-experiencing (B = −.001, *p* = .237). Problem-solving appraisal also did not moderate the relationship between social media integration and PCL-5 symptom severity (B = .006, *p* = .298), re-experiencing (B = .003, *p* = .098), avoidance (B = .000, *p* = .716), negative alterations in cognitions and mood (B = .001, *p* = .573), and hyperarousal (B = .001, *p* = .405). All of the significant moderating effects remained significant after the false discovery rate procedure.

Table 4 summarises the nature of the moderation effect at different levels of social support (conditional effects), while Fig 2 provides a graphical representation of the moderation effect.

**Table 4:** The effects of social media integration on PCL-5 symptom severity at different levels of social support.

Value of the moderator	B	SE	95% CI		p
			LL	UL	
PCL-5 symptom severity					
1 *SD* below the mean	.73	.10	.53	.93	<.001
At the mean	.57	.08	.43	.72	<.001
1 SD above the mean	.42	.10	.21	.62	<.001
Avoidance					
1 *SD* below the mean	.08	.01	.05	.11	<.001
At the mean	.06	.01	.04	.08	<.001
1 SD above the mean	.04	.01	.01	.03	.011
Negative alterations					
1 *SD* below the mean	.27	.04	.19	.35	<.001
At the mean	.21	.03	.15	.27	<.001
1 SD above the mean	.14	.04	.07	.22	<.001
Hyperarousal					
1 *SD* below the mean	.22	.03	.16	.29	<.001
At the mean	.17	.02	.13	.22	<.001
1 SD above the mean	.13	.03	.06	.19	<.001

**Fig 2:**
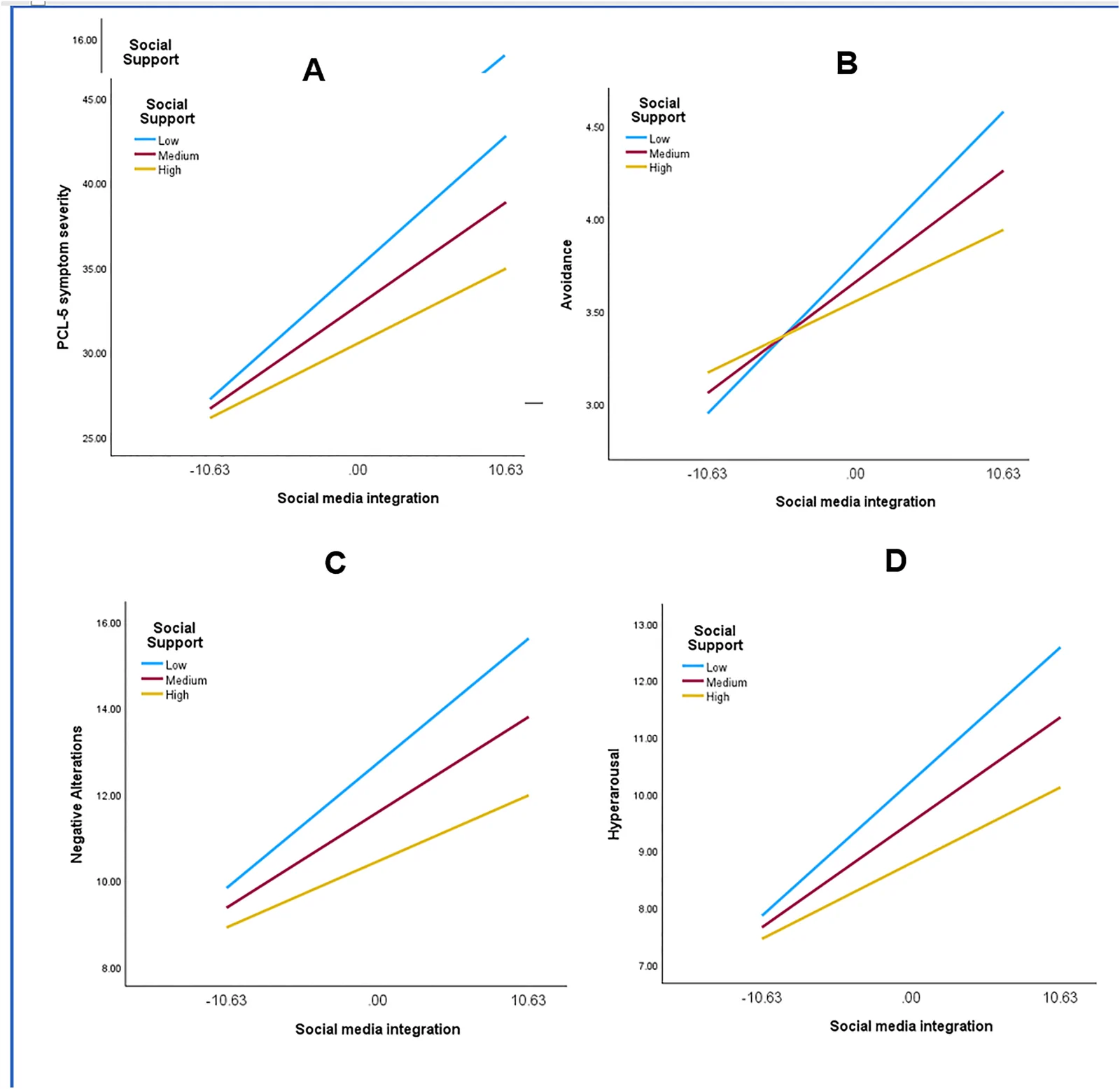
The moderating role of social support. Fig 2A = the moderating role of social support in the relationship between social media use and PCL-5 symptom severity, Fig 2B = the moderating role of social support in the relationship between social media use and avoidance, Fig 2C = the moderating role of social support in the relationship between social media use and negative alterations in cognition and mood, Fig 2D = the moderating role of social support in the relationship between social media use and hyperarousal.

Table 4 indicates that the unstandardised coefficient for the relationship between social media use and PCL-5 symptom severity was greater at low levels of social support (1*SD* below the mean: B = .73) than at high levels of social support (1*SD* above the mean: B = .42), thus signifying that the relationship between social media integration and PCL-5 symptom severity was stronger at lower levels of social support. The same applies to avoidance (1*SD* below the mean: B = .08; 1*SD* above the mean: B = .04), negative alterations in cognition and mood (1*SD* below the mean: B = .27; 1*SD* above the mean: B = .14), and hyperarousal (1*SD* below the mean: B = .22; 1*SD* above the mean: B = .13), where the relationships between social media integration and avoidance, negative alterations in cognition and mood and hyperarousal were stronger at lower levels of social support than at higher levels. These results are confirmed by the plots of the interaction between social media integration and social support in Fig 2.

The regression lines in Fig 2A–2D are noticeably steeper at a low level of social support than at a high level of social support, thus confirming that high levels of social media integration were more strongly associated with PCL-5 symptom severity, avoidance, negative alterations in cognition and mood and hyperarousal, respectively, at low levels of social support than at higher levels of social support.

## Discussion

The study aimed to investigate the role of appraisals of problem-solving ability and social support in the cross-sectional association between social media integration and PCL-5 symptom severity. There were several significant findings. First, social media integration was significantly negatively associated with problem-solving appraisal and significantly positively associated with social support. This suggests that less favourable appraisals of problem-solving ability were associated with higher SMUIS scores, reflecting stronger social media integration and emotional connection. One possible interpretation is that individuals who feel less confident in their ability to handle everyday difficulties may be more likely to turn to social media for distraction, avoidance, or emotional regulation. This finding is in line with existing research on self-confidence and self-efficacy, which indicates that lower levels of these constructs are associated with increased social media use [42].

By contrast, the positive cross-sectional association between social media use integration and perceived social support indicates that students for whom social media is more strongly embedded in their everyday routines and social lives also tend to perceive higher levels of support from others. This may reflect the capacity of social media platforms to facilitate connection, communication, and access to supportive social networks [21].

Second, social media integration was significantly positively associated with PCL-5 symptom severity. In other words, higher levels of social media integration were associated with higher levels of PCL-5 re-experiencing, avoidance, negative alterations in cognition and mood, and hyperarousal symptoms. Social media integration may increase exposure to distressing or emotionally activating content, including trauma-related reminders or distressing material that intensifies negative affect and emotional arousal. For individuals already vulnerable to trauma-related distress, such exposure may contribute to intrusive thoughts, emotional dysregulation, and heightened physiological activation [11].

The cross-sectional association may also reflect the role of social media as a maladaptive coping strategy. Individuals experiencing trauma-related symptoms may turn to social media as a form of distraction or avoidance; however, this pattern of use may ultimately maintain or exacerbate symptoms rather than alleviate them [43,44]. In this sense, social media may function as a context in which avoidance-based coping is reinforced.

Third, problem-solving appraisal had significant negative direct effects on all PCL-5 symptom clusters, except avoidance. This means that individuals who perceived themselves as more capable of effectively managing problems and coping with challenges reported fewer PCL-5 intrusion, negative alterations in cognition and mood, and hyperarousal symptoms. This finding underscores the protective role of problem-solving appraisal and suggests that it may serve as an important cognitive resource in mitigating trauma-related symptoms. It also affirms the findings in the existing literature on the role of positive appraisal style in the relationship between stress and mental health outcomes [15,45].

Fourth, consistent with the broader literature, higher levels of perceived social support were associated with lower levels of negative alterations in cognition and mood, and lower levels of hyperarousal. Supportive relationships may help to reduce feelings of isolation, shame, helplessness, and hopelessness, while also fostering a greater sense of emotional security and interpersonal connectedness. In this way, social support may mitigate some of the maladaptive cognitions commonly associated with PCL-5 symptoms. It may also contribute to a sense of safety and regulation, thereby reducing symptoms of heightened arousal [46,47].

However, social support alone may be less effective in addressing symptoms such as intrusive re-experiencing. Such symptoms are more directly tied to disturbances in trauma memory processing and involuntary memory activation. Accordingly, while social support may buffer against distress and facilitate coping, it may not be sufficient on its own to address the underlying memory-related processes that give rise to recurrent intrusive thoughts, images, or flashbacks [16,48].

Fifth, problem-solving appraisal partially mediated the relationship between social media integration and PCL-5 symptom severity across all symptom clusters. This suggests that the association between stronger social media integration and emotional connection and higher trauma-related symptomatology operates, at least in part, through diminished perceptions of one’s problem-solving ability. Other processes are likely to contribute to the cross-sectional association between social media integration and PCL-5 trauma symptoms. Even so, the consistency of the mediating effect across symptom domains reinforces the broad relevance of problem-solving appraisal as a cognitive mechanism for understanding the link between social media integration and PCL-5 symptom severity.

Finally, social support moderated the relationship between social media integration and overall PCL-5 symptom severity, negative alterations in cognition and mood, and hyperarousal. This suggests that supportive interpersonal relationships may buffer the adverse psychological effects of social media use by enhancing feelings of safety, belonging, and emotional regulation. These protective effects may be particularly relevant for mood-related and arousal-related symptoms, which are more amenable to interpersonal reassurance. Similar results regarding the role of social support in the cross-sectional association between social media use and mental health outcomes have been documented [49,50].

The study has implications for theory and clinical practice. At a theoretical level, the study supports stress and coping frameworks by demonstrating that trauma-related distress, in the context of social media integration, is influenced by both internal coping appraisals and external relational resources. The direct and mediating roles of problem-solving appraisal suggest that interventions aimed at strengthening coping confidence and problem-solving ability may help reduce vulnerability to PCL-5 symptomatology among young people with high levels of social media engagement. This points to the value of incorporating problem-solving skills training, cognitive restructuring, and coping-focused interventions into trauma-informed mental health support.

The moderating role of social support further suggests that strengthening supportive social networks may buffer some of the harmful effects of social media integration, particularly in relation to negative mood-related symptoms and hyperarousal. Given that social media use was associated with higher PCL-5 symptomatology across all symptom clusters, clinicians, educators, and university support services may need to be more attentive to the role of digital environments in trauma-related distress. Psychoeducation could be beneficial in promoting recognition that engagement with social media may serve as an avoidance strategy, a source of triggering content, or a context that amplifies distress.

The findings of the study should be interpreted in the context of several limitations. All variables were assessed at a single time point, preventing the establishment of temporal ordering. The indirect associations therefore do not demonstrate causal mediation, and the interactions do not establish that perceived social support protects against subsequent symptoms. Reverse and reciprocal associations remain possible, and unmeasured factors could also account for some of the observed associations.

All measures were self-reported and administered within the same questionnaire. Recall difficulties, social desirability, current mood, and individual response tendencies may have influenced responses. The shared measurement method introduces the possibility of common-method variance, which may have affected the observed associations. However, its magnitude and direction cannot be established from the present data. Future research would benefit from incorporating multi-method approaches, including clinician-administered assessments and behavioural indicators of social media use, to strengthen the validity of the findings.

The study was conducted within a single geographic area, which may limit the generalisability of the findings to other settings or populations. Replication across more diverse geographic and sociocultural contexts is needed to determine the broader applicability of the results. Problem-solving appraisal partially mediated the relationship between social media integration and PCL-5 symptom severity, indicating that additional explanatory variables are likely involved. Future research should therefore examine a wider range of potential mediating mechanisms to develop a more comprehensive understanding of these associations.

The study did not collect information on the specific social media platforms used by participants or on behavioural indicators such as frequency and duration of use. Consequently, it was not possible to determine whether the observed cross-sectional associations differed across platforms or according to the amount of time participants spent on social media. The findings should therefore be interpreted as reflecting the integration of social media into participants’ everyday routines and social lives, together with their emotional connection to it, rather than the frequency, duration, or platform-specific nature of their use.

An important limitation concerns the administration and interpretation of the PCL-5. The study did not assess whether participants had experienced a potentially traumatic event meeting DSM-5 Criterion A, and no trauma-exposure instrument or screening questions were administered. Participants’ responses were also not anchored to a specified index event. Consequently, it was not possible to establish whether participants had experienced a qualifying event, determine the frequency or types of events experienced, or ascertain whether the reported symptoms were attributable to such an event. No participants were excluded on the basis of Criterion A exposure, and analyses restricted to participants with a qualifying event could not be conducted. The findings must therefore be interpreted as cross-sectional associations with PCL-5 symptom severity and not as evidence concerning PTSD, probable PTSD, or verified post-traumatic stress symptoms. Future research should assess Criterion A exposure using an appropriate trauma-exposure instrument, identify an index event, and anchor PCL-5 responses to that event.

The approximate response rate was 12.3%. Although the Registrar randomly selected students to receive invitations, participation was voluntary, leaving the achieved sample susceptible to non-response and self-selection bias. Respondents may have differed from nonrespondents in digital engagement, psychological distress, or interest in the study topic. Information about nonrespondents was unavailable, preventing assessment of these differences. The required-response format eliminated item-level missing data among submitted questionnaires but did not eliminate the possibility of survey abandonment or participation bias.

Finally, participants attended a single university, and most were undergraduate students. Representation from different provinces does not make the sample representative of South African university students or young adults more broadly. Race or ethnicity, year of study, and academic discipline were not collected, further limiting assessment of representativeness. The exploratory, post hoc status of the moderation analyses also warrants caution, particularly given the multiple outcomes examined. These findings require replication in independent samples.

Future research should use longitudinal designs with repeated assessments of social media integration, problem-solving appraisal, perceived social support and symptom severity. Designs incorporating appropriately spaced assessments would permit investigation of whether changes in social media integration precede changes in appraisal variables and subsequent symptoms, and would also test reverse and reciprocal associations. Such designs would strengthen temporal inference.

Future studies should also assess active communication, content creation, passive browsing, duration of use, and the emotional and interpersonal context of these activities. Research has demonstrated variation in associations between social media use and well-being, supporting attention to person-specific patterns [51,52].

Additional cognitive and interpersonal processes warrant prospective investigation. Research on online support-seeking and co-rumination provides a basis for examining whether repetitive discussion of difficulties differs from constructive support-seeking in its relationship with later symptoms [53]. Future longitudinal studies should examine how rumination and emotion regulation relate to social media integration and PCL-5 symptom severity over time.

Future investigations of PTSD should assess Criterion A exposure, identify an appropriate index event, and anchor PCL-5 responses to that event. Clinical interviews in a subsample could provide additional evidence concerning diagnostic status. Multisite recruitment across different institutions, together with more complete demographic information and procedures to evaluate non-response, would improve generalisability. Finally, hypotheses, mediator and moderator roles, primary outcomes and approaches to multiple testing should be specified prospectively. Independent replication should precede claims that strengthening problem-solving appraisal or perceived social support would alter the relationship between social media integration and symptom severity.

## Conclusion

The present study contributes to the growing literature on social media integration and post-traumatic stress symptomatology by demonstrating that stronger social media integration was cross-sectionally associated with higher levels of PCL-5 scores, while problem-solving appraisal and perceived social support emerged as important protective factors. Taken together, these findings highlight the importance of considering both cognitive and interpersonal resources in understanding the psychological consequences of social media use and suggest that interventions aimed at strengthening coping appraisals and supportive social connections may help reduce vulnerability to trauma-related symptoms.
